# Do residents of food deserts express different food buying preferences compared to residents of food oases? A mixed-methods analysis

**DOI:** 10.1186/1479-5868-9-41

**Published:** 2012-04-10

**Authors:** Jason Block, Ichiro Kawachi

**Affiliations:** 1 Department of Society, Human Development and Health, Harvard School of Public Health, 401 Park Drive, Room 445-C, Boston, MA, 02215, USA; 2 University of Wisconsin-Milwaukee, Joseph J. Zilber School of Public Health, Alumni House 327, P.O. Box 413, Milwaukee, WI, 53201-0413, USA; 3 Department of Population Medicine, Harvard Medical School, 133 Brookline Avenue, 3rd Floor, Boston, MA, 02215, USA; 4 Department of Society, Human Development, and Health, Harvard University School of Public Health, 677 Huntington Avenue, Boston, MA, 02115, USA

**Keywords:** Food access, Food desert, Food oasis, Food buying practices, Concept mapping, Low-income

## Abstract

**Background:**

Many people lack access to food stores that provide healthful food. Neighborhoods with poor supermarket access have been characterized as “food deserts” (as contrast with “food oases”). This study explored factors influencing food buying practices among residents of food deserts versus food oases in the city of Boston, USA.

**Methods:**

We used the mixed-methods approach of concept mapping, which allows participants to identify, list, and organize their perceptions according to importance. Resulting maps visually illustrate priority areas.

**Results:**

Sixty-seven low-income adults completed the concept mapping process that identified 163 unique statements (e.g. relating to affordability, taste, and convenience) that influence food buying practices. Multivariate statistical techniques grouped the 163 statements into 8 clusters or concepts. Results showed that average cluster ratings and rankings were similar between residents of food deserts and food oases.

**Conclusions:**

The implication of this study pertains to the importance of community resources and emergency food assistance programs that have served to minimize the burden associated with hunger and poor food access among low-income, urban populations.

## Background

Neighborhood differences in the local food environment have been increasingly implicated in the rising prevalence of obesity in the United States. Studies have reported an association between neighborhood food environment and obesity prevalence in the United States. Maddock [1] found a positive association between fast food restaurant density and obesity prevalence. A study by Inagami and colleagues [2] showed a higher body mass index (BMI) among those who shopped in disadvantaged neighborhoods – defined by lower sociocenomic status. Additionally, two studies by Morland et al., [3,4]showed that obesity prevalence was lower in areas where supermarkets were located compared to areas with grocery stores or fast food restaurants. The presence of a supermarket is often viewed as the “gold standard” in food access given the lower costs, increased quantity and improved quality of food items available at chain supermarkets compared to their non-chain counterparts. Despite support for the association between supermarket access and obesity prevalence, there are neighborhoods at increased risk for diet-related health outcomes due to the absence of a supermarket. Particular attention has been paid to neighborhoods that lack a supermarket – termed “food deserts” -- given the convenience that shopping locally affords residents [5]. These areas devoid of supermarkets have been described by Walker et al. [6] in a comprehensive literature review of food deserts in the United States. The review identified four thematic areas of ongoing research, including: (a) local availability of supermarkets; (b) racial/ethnic disparities in food access; (c) socioeconomic disparities in food access; and (d) differences in chain versus non-chain stores 6. Disparities in the neighborhood food environment have prompted research exploring the impact of the local food environment on food purchasing behavior. Studies exploring the intersection between the neighborhood food environment and food purchasing behavior have showed that living in a disadvantaged neighborhood is associated with greater consumption of energy-dense foods [7,8]. Research by Cummins and Macintyre [9]suggests that the neighborhood food environment has an independent effect on diet by influencing food buying practices. Similar findings were observed in studies that showed a positive association between proximity to a supermarket and individual diet [10,11] and studies that found a positive association between the presence of a neighborhood supermarket and increased consumption of fruits and vegetables [12-14]. On the other hand, fewer nutrient-dense food options available at neighborhood food stores has been associated with lower consumption of these foods by local residents [15]. However, one of the challenges in this field of research is the extent to which causality can be attributed to local variations in the food environment [16]. That is, even if variations in the quality of the local food environment can be mapped on to local variations in nutritional quality and obesity prevalence, it does not necessarily prove that one caused the other. A correlation between local food environments and obesity rates could simply reflect the food preferences of residents. Alternatively, confounding by unmeasured variables may be explaining the correlations observed between food environments and obesity rates. Thus, from a supply-side perspective, grocery owners are unlikely to stock fresh produce on their shelves in neighborhoods where residents are unlikely to demand them. However, it is important to note that interactions between the neighborhood food environment, diet quality and subsequent health outcomes is multifocal in nature whereby causation involves not one pathway, but multiple pathways.

Few studies have directly examined the food preferences of residents in disadvantaged areas. Eikenberry and Smith conducted a study to identify motivators, barriers and promoters of healthy eating among select rural and urban food deserts in Minnesota [17]. Study participants expressed a lack of desire to consume fresh produce even when they were made available [18]. Similarly, Walker and colleagues found that low-income residents of a food desert did not prefer to consume fruits and vegetables due to their aftertaste [19]. While these findings are not generalizable to residents of other food deserts, it begs further exploration of the influence of food preference.

Food preferences have been described in the literature as influencing food buying and consumption practices. Locher et al., [20] explored how psychosocial factors (motivation, perceived barriers, social demographics, and dietary quality) influence food choices among homebound older adults. Findings from this study suggested that sensory factors, specifically taste and aesthetics, were primary motivators involved in food choice [20,21]. Other studies have explored how cultural attitudes [22] and perceptions of healthy eating and the food environment influence food choice [23,24]. These studies indicated that taste, texture, and appearance were more important for food choices than nutritional content [22]. This evidence supports the argument that food preference is a strong predictor of food consumption and can serve to promote (or deter) healthy eating.

In addition to food preferences being strong predictors in food buying and consumption practices, consumers often express a preference for type of store patronized. Many paramount studies exploring consumer food shopping behavior have identified factors including location, variety of goods, store loyalty and store policies as reasons why a particular store was patronized over another [25-27]. In the case of neighborhood convenience and corner stores, it is believed that clerk personality and established friendships between store owners and patrons foster store loyalty [25]. This is especially salient in neighborhoods where chain supermarkets are not located. The implication is that residents of these neighborhoods without a supermarket may view the role of the independent store as an anchor within the community thereby encouraging patronage. Subsequently, the food items available within these stores are important for considering food purchasing and consumption behaviors.

Given the influence the neighborhood food environment has on food buying practices among local residents, we sought to explore factors influencing food buying practices among low-income residents with differential supermarket access in the present study. We conducted a mixed-method approach based on concept mapping [28,29] to ascertain the food purchasing practices and preferences of two contrasting neighborhoods in the city of Boston, MA. We hypothesized that differences in food buying practices and food preferences would be identified based on residing in a neighborhood categorized a food desert compared to a food oasis.

## Methods

The geographic unit of analysis for this study was the zip code, which served as our model of a neighborhood. Zip codes in the United States do not represent a geographical area, per se, but are a network of roads and addresses used for the purpose of mail delivery [30,31]. Therefore, there is great variability in the size of a zip code. For example, Grubesic [30] reported an average zip code size in the state of Wyoming to be 1,430 square kilometers (km^2^) compared to 12.8 km^2^ for an average zip code size in the state of New Jersey. Despite this variability, zip codes are frequently used as the unit of analysis in research given their geographical context [30]. We selected four zip codes for the study – two characterized as food deserts and two as food oases. The average land area for the food deserts was 8.0 km^2^ (3.1 square miles) compared to 3.6 km^2^ (1.4 square miles) for the food oases [32]. We defined a food desert as a zip code that does not have a chain supermarket within 0.5 miles of the center of the zip code. Conversely, we defined a food oasis as a zip code that does have a chain supermarket within 0.5 miles of the center of the zip code. The definition of the buffer zone is consistent with the literature, with 0.5 miles commonly perceived as a reasonable walking distance for an adult to carry home bags of groceries [33]. To identify Boston zip codes selected for the study, two approaches were used. First, we examined the online yellow pages (http://www.yellowpages.com) to identify zip codes that do not have supermarket access. This is consistent with other studies that utilized yellow pages to identify resources in proximity to a specific unit of area [34,35]. Second, we examined U.S. census data to characterize each zip code based on median household income. The selected zip codes had some of the lowest median household incomes for the city of Boston. We matched the selected food deserts with comparable food oases on various demographic characteristics including median household income, age, race/ethnicity, and educational attainment.

### Recruitment

We placed advertisements in popular print and online newspapers. Print advertisements ran on three non-consecutive days over a period of 3 weeks. Online advertisements appeared continuously for a period of three weeks. The advertisement included the zip codes of interest and a phone number that potential participants were asked to call for additional information. Participants were expected to meet three inclusion criteria: 1. At least 18 years of age; 2. A current resident of one of the study zip codes; and 3. A resident of the zip code for the previous 12 months. If all inclusion criteria were met and the potential participant was interested in participating in the study, a consent form was mailed for review prior to the start of the study. The study was approved by the Institutional Review Board of the Harvard School of Public Health.

### Concept mapping methodology

Concept mapping is a mixed methods approach originally used in program evaluation [36] but has since been used in the social sciences for examining other complex phenomena. Concept mapping studies have explored a variety of topic areas including the relationship between neighborhoods and mental health [37] and perceptions of neighborhood influences on health among immigrant populations [38]. The concept mapping process includes six steps: 1) Preparation, 2) Brainstorming, 3) Sorting and rating, 4) Data Analysis, 5) Interpretation, and 6) Utilization of concept maps. The concept mapping process has been extensively described in the literature [29,35,39,40]. For this study, the six steps of the concept mapping process occurred over a period of three non-consecutive days. We conducted three concept mapping sessions in each of the four zip codes with the same participants in each session. Since each session built on the participant discussions during the previous sessions, we asked participants to attend all three sessions conducted in their respective zip code. Sixty-seven participants completed the concept mapping process. There was no loss to follow-up over the three sessions. Thirty-five participants were residents of the food desert and 32 from the food oasis.

Day 1: Brainstorming Session – During this 2 h session, we asked participants to freely generate words or short phrases in response to the focus statement: “What things, good or bad, influence your food buying practices?” We defined the phrase “food buying practices” as “where you buy food, the types of food you buy, and when you buy food.” Participants responded to the focus statement using a round-robin approach. After an exhaustive list was generated, participants went through the list to consolidate statements they thought were too similar or overlapping. For example, participants consolidated the statements “billboards” and “food advertisement” into the statement “advertisements.” Participants received a $20 gift card for completing this session.

Day 2: Sorting and Rating Session – Participants returned for the second session 3 weeks after the first concept mapping session (brainstorming session). Each participant received a stack of note cards with a unique statement generated during the previous brainstorming session written on each card. Participants worked independently to sort the cards into piles that “make sense to you.” After sorting the cards, participants assigned each pile a label or name that represented the contents of the pile. For example, one participant may have sorted the statements “fuel perks (gas discount),” “what stores are near where I live,” “T-Accessible (public transportation),” and “gypsy cabs (unlicensed taxis)” into the same pile and labeled the pile “Transportation.” A second activity was to rate how important each statement was to influencing food buying practices according to a Likert scale (1 = not at all important to 5 = extremely important). Participants received a $25 gift card for completing this session.

Day 3: Interpretation Session – Two weeks after the second concept mapping session, the same participants returned to interpret the concept maps generated in the second session. In small groups, participants were given flip chart paper and markers and asked to diagram how the statements generated within a cluster influenced food buying practices. In other words, participants were asked to generate a visual depiction of how the statements influenced food buying practices. Participants received a $30 gift card for completing this session. For attending all three sessions, participants received a total of $75 in gift cards.

## Data analysis

We undertook three main analyses. First, we entered results from the sorting and rating step into specialized software, Concept Systems, Inc. [41] for analysis. A benefit to using Concept Systems software is that data are entered and analyzed in real time while participants complete other study activities. The second analysis utilized multidimensional scaling which took individual data across all participants and using a similarity matrix noted similarities and dissimilarities in how the data were sorted. An aggregate group product was produced in the form of a *point map*. A point map visually depicts how each statement was sorted with respect to the other statements for the entire group thereby incorporating each participant’s sort data. Each point on the point map represents one of the unique statements generated during the brainstorming session. Points that are in close proximity were sorted more frequently by participants compared to points that are further apart.

The third analysis, *hierarchical cluster analysis*, partitioned the point map into clusters representing unique concepts or ideas. The final number of clusters selected was identified by participants as the appropriate number of clusters that represented their perceptions of factors that influence their food buying practices. Another type of concept map produced, *a pattern match*, is a ladder graph representation that allows comparisons to be made between groups. A horizontal rung of the ladder corresponds to a perfect correlation between the two groups being compared. For this study, a comparison was made between participants of the food desert and participants of the food oasis. A Pearson product–moment correlation was calculated to represent the correlation between the two groups.

## Results

### Participant characteristics

The sample consisted of 67 participants. The median age was 47 years. The sample was predominantly female (56.7%) and African American (67.2%). More than half of the sample did not own a personal car and found it difficult to find a ride (52.2%). Table 1 displays additional participant characteristics.

**Table 1:** Participant Characteristics by Food Desert Status

**Zip code characteristic**	**Food desert**	**Food oasis**	**Total**
Total number of participants (%)	35 (52.2)	32 (47.8)	67.0
Age			
Median age (years)	47.0	47.3	47.0
Range	28–66	32–61	28–66
Sex – n (%)			
Male	15 (42.9)	14 (43.8)	29 (43.3)
Female	20 (57.1)	18 (56.2)	38 (56.7)
Race & Ethnicity – n (%)			
African American	20 (57.1)	25 (78.1)	45 (67.2)
Caucasian	9 (25.7)	2 (6.3)	11 (16.4)
Hispanic/Latino	3 (8.6)	2 (6.3)	5 (7.5)
Other	3 (8.6)	3 (9.4)	6 (8.9)
Car ownership			
Do not own car & hard to find a ride	18 (51.4)	17 (53.1)	35 (52.2)
Do not own car & able to find a ride	9 (25.7)	10 (31.3)	19 (28.4)
Own car	8 (22.9)	5 (15.6)	13 (19.4)
Nearest bus stop – n (%)			
<1 block	10 (28.6)	14 (43.8)	24 (35.8)
1–2 blocks	12 (34.3)	5 (15.6)	17 (25.4)
3–4 blocks	8 (22.9)	8 (25.0)	16 (23.9)
5 or more blocks	5 (14.2)	5 (15.6)	10 (14.9)
Number of different bus routes near home – n (%)			
1–2 routes	16 (45.7)	17 (53.1)	33 (49.2)
3–4 routes	9 (25.7)	8 (25.0)	17 (25.4)
5 or more routes	10 (28.6)	7 (21.9)	17 (25.4)
Bus frequency – n (%)			
< 15 minutes	9 (25.7)	10 (31.3)	19 (28.4)
Every 15–30 minutes	17 (48.6)	16 (49.9)	33 (49.3)
Every 30–45 minutes	6 (17.1)	3 (9.4)	9 (13.4)
> 45 minutes	3 (8.6)	3 (9.4)	6 (8.9)

**Figure 1:**
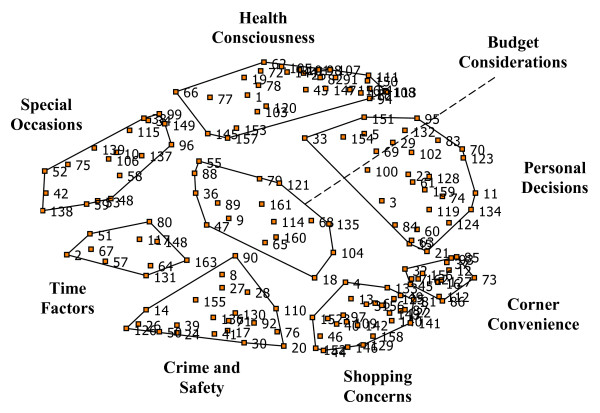
Point Cluster Map of a 8-Cluster Solution.

The two food deserts generated 110 and 182 unique or non-overlapping statements. The two food oases generated 147 and 171 unique statements. All of the statements generated from the four groups were combined into one list. Participants engaged in a process of removing duplicate entries and consolidating similar statements to generate a final master list. The final master list included 163 unique or non-overlapping statements. Examples of unique statements included in the final master list were “appearance of food,” “brand names,” “ease of theft,” “microwaveable foods” and “preference.” The 163 statements were partitioned into 8 clusters. Participants were initially presented with a 12 cluster solution map. Participants stated that the 12 clusters, or unique concepts were overlapping and did not accurately reflect their perceptions. After using the specialized software to decrease the number of clusters, we presented a final 8 cluster solution map agreed upon and named by participants. Figure 1 illustrates the point cluster map with the names of each cluster given by participants. Statement numbers represented as points on the map presented in 1 can be linked to Table 2 to identify the exact statement name. Overlapping points on the map illustrate areas with a high degree of agreement in participant perceptions of the relationship between statements. It may appear that specific statements do not fit into a certain cluster. Two explanations can be offered to address this seemingly inconsistency. First, the way participants perceived the statement influenced how the statements were sorted together. Second, statements participants perceived as not fitting nicely into one pile may have been sorted with other statements that were considered “leftovers.” However, it is important to note that a spanning analysis, a type of analysis used to identify how statements were sorted relative to other statements, were performed to explore these inconsistencies. The results (figures not shown) suggest that participants were consistent in how these statements were sorted, thereby suggesting a perspective based on lived experiences outside the understanding of the researchers.

**Table 2:** Average Cluster and Statement Ratings by Food Desert Status

**Cluster Name and Statements (Statement ID Number)**	**Influences Food Buying Practices**		**Cluster Name and Statements (Statement ID Number)**	**Influences Food Buying Practices**	
	**Food Desert**	**Food Oasis**		**Food Desert**	**Food Oasis**
**HEALTH CONSCIOUSNESS**	**Moderate**	**High**	**PERSONAL DECISIONS**	**Moderate**	**High**
Taste (147)	High	High	Freshness (70)	High	High
Knowing how to cook (94)	High	High	Cost (35)	High	High
How food is prepared (82)	High	High	What your needs are (159)	High	High
Personal health (116)	High	High	Appearance of food (11)	High	High
Portion size (118)	High	High	Quality (123)	High	High
Food preparation time (66)	High	High	Food handling (63)	High	High
High cholesterol (78)	High	High	Labels (95)	High	High
Ingredients in food (91)	High	High	Quantity of items (124)	High	High
Texture of food (150)	High	High	Preference (119)	High	High
More health conscious (107)	High	High	How food is stored (83)	High	High
Eating balanced meals (54)	High	High	Seasonings I need to cook with (132)	High	High
Nutritional content (111)	High	High	How much I can carry (84)	High	High
Natural food (108)	Moderate	High	Making food stretch (102)	High	High
A lot of sodium in foods (1)	Moderate	High	Samples (128)	Moderate	High
Fat content (62)	High	High	The cost of eating healthy makes me buy less (151)	Moderate	High
Sugar content (144)	High	High	Farmer’s markets (61)	High	High
Diet (43)	High	High	Buy in bulk (21)	Moderate	High
Calorie intake (25)	High	High	Freezer space (69)	Moderate	High
What foods make you tired (157)	Moderate	High	Cook large quantities and eat for the week (33)	High	High
Losing weight (98)	High	High	Able to cook large quantities to last a few days (5)	Moderate	High
Healthy eating is too difficult (77)	Moderate	High	Family-sized packages (60)	Moderate	High
Organic foods (113)	Moderate	High	Grocery lists (74)	High	Moderate
Genetically modified foods (72)	Moderate	Moderate	Cheaper to cook than eat at fast food restaurants (29)	High	Moderate
Modify prepared foods so healthier (105)	Moderate	Moderate	Variety of prepared food (154)	Moderate	Moderate
Break down large quantities into smaller servings (19)	High	Moderate	Able to buy ethnic foods (3)	Low	Moderate
Make your own foods instead of foods in a jar (101)	Moderate	Moderate	Shop everyday to have fresh food (134)	Low	Moderate
Use of food with medications (153)	High	Moderate	Buy packaged food and jazz it up (22)	Moderate	Moderate
Processed foods (120)	Low	Moderate	Make weekly menus (100)	Moderate	Moderate
Microwaveable foods (103)	Moderate	Moderate			
Supplement cooked food with restaurant food (145)	Moderate	Moderate			
Supplement cooked food with restaurant food (145)	Moderate	Moderate			
**TIME FACTORS**	**Moderate**	**Moderate**	**SPECIAL OCCASIONS**	**Moderate**	**Moderate**
Food recalls (67)	High	High	Lifestyle (96)	High	High
Expiration date (57)	High	High	Family upbringing (59)	Moderate	High
Household preference (80)	Moderate	High	Family influence (58)	Moderate	High
Season (131)	Moderate	High	Loss of appetite (99)	Moderate	High
Food Industry (64)	High	Moderate	Mood swings (106)	Moderate	High
Television (148)	Moderate	Moderate	Guests/Company (75)	Moderate	High
Word of mouth (163)	Moderate	Moderate	Special occasions (139)	Moderate	High
Pets need to eat (117)	Moderate	Moderate	Don’t go shopping when you’re hungry (48)	Moderate	High
Easier to buy at fast food restaurants (51)	Low	Moderate	Participation in research studies (115)	High	High
A treat is eating at a high end restaurant (2)	Low	Moderate	Cravings (38)	Moderate	Moderate
			Temptation (149)	Moderate	Moderate
			Cooking fatigue (34)	Moderate	Moderate
			Depression (42)	Moderate	Moderate
			Easier to buy ready-made food (52)	Low	Moderate
			Social circles (138)	Moderate	Moderate
			Eating at restaurants make me buy less at stores (53)	Low	Moderate
			Anxiety (10)	Moderate	Moderate
			Short lunch period, so I eat fast foods (137)	Low	Moderate
**CRIME AND SAFETY**	**Moderate**	**Moderate**	**BUDGET CONSIDERATIONS**	**Moderate**	**Moderate**
Inflation (90)	High	High	Income (89)	High	High
Amount of money you have to spend (8)	High	High	Education (55)	High	High
Being overcharged repeatedly (17)	High	High	When you get your Social Security check (161)	High	High
Schedule (130)	High	High	Food pantries (65)	Moderate	High
Rodents are common around food (126)	High	High	Household composition (79)	Moderate	High
Bad experiences (14)	Moderate	High	Dollar menus (47)	Moderate	High
Weather (155)	High	Moderate	Minimum purchase required for using certain payment (104)	Moderate	High
No one-stop shopping in “the hood” (110)	Moderate	Moderate	When you get food stamps (160)	Moderate	Moderate
Buy 2 get $1 off can be a trick (20)	Moderate	Moderate	Professional recommendation (121)	Moderate	Moderate
Items without a price tag (92)	Low	Moderate	Brand names (18)	Moderate	Moderate
Cheaper foods in the suburbs (28)	Moderate	Moderate	Impulse (88)	Moderate	Moderate
Customers get into fights (41)	Moderate	Moderate	Anticipating what food pantry will distribute (9)	Moderate	Moderate
Crime (39)	High	Moderate	Overwhelmed by options (114)	Moderate	Moderate
Gypsy cabs (76)	Low	Moderate	Food trucks on the side of the road have fresh and cheap food (68)	Moderate	Moderate
Celebrity endorsements (27)	Low	Moderate	Country where food is made (36)	Low	Moderate
Fuel perks (71)	Moderate	Moderate	Shopping when tired (135)	Low	Moderate
Cab fare (24)	Moderate	Moderate			
Shopping with kids or grandkids (136)	Low	Low			
Child care at the store (30)	Low	Low			
Ease of theft (50)	Low	Low			
Can steal under $250 without being arrested (26)	Low	Low			
**SHOPPING CONCERNS**	**Moderate**	**High**	**CORNER CONVENIENCE**	**High**	**High**
Store crowding (142)	High	High	Good sanitation at the meat counter (73)	High	High
Store cleanliness (141)	High	High	Availability of food (12)	High	High
Double coupons (49)	High	High	Discount card (45)	High	High
T-Accessible (146)	Moderate	High	Bargains (16)	High	High
Distance (46)	High	High	Sales (127)	High	High
What stores are near where I live (158)	High	High	Know what to buy at different stores (93)	High	High
Scent of store (129)	High	High	How neat the store is (86)		
Store hours (143)	High	High	One-stop shopping (112)	Moderate	High
Transportation (152)	High	High	Coupons (37)	High	High
Enough cashiers (56)	High	High	Convenience (32)	High	High
Reputation of store (125)	High	High	Where gift cards are for (162)	High	High
Customer service (40)	High	High	How much time I have to shop (85)	High	High
Availability of sale items (13)	High	High	What can fit in the shopping cart (156)	Moderate	High
How comfortable you feel at the store (81)	High	High	Amount gift cards are for (7)	High	High
Different locations have different prices and selection (44)	High	High			
Long lines (97)	Moderate	High			
Neighborhood of the store (109)	Moderate	High			
Circulars (31)	High	Moderate			
Advertisements (6)	Moderate	Moderate			
Stop & Shop and Shaw’s have different departments (140)	Moderate	Moderate			
How well you know the layout of the store (87)	High	Moderate			
Promotions (122)	High	Moderate			
Self check-out (133)	Moderate	Moderate			
Bag your own groceries (15)	Low	Moderate			
Buying grocery bags (23)	Low	Low			
Able to buy religious foods (4)	Low	Low			

Note: The 163 statements are presented within their clusters (capitalized and bolded) and the parenthetical numbers refer to the actual statement number and can be used to link the table information to Figure 1. The numbers do not have any substantive meaning. Ratings represent how strongly each statement influences food buying practices. Ratings between 1.29-2.50 are designated low, 2.51-3.72 designated moderate and 3.73-4.93 designated high.

### Average cluster and statement ratings

Participants rated how important each statement was to influencing food buying practices on a scale of 1 (not at all important) to 5 (extremely important) during the sorting and rating step of the concept mapping process. Table 2 outlines the average statement and cluster ratings. The table is organized by cluster with the cluster name in bold type. Underneath each cluster label are the statements that comprised that cluster. The number besides each statement label (in parenthesis) represents the statement number that can be used to link each statement with the location on the point cluster map (Figure 1). The two columns next to the cluster label and corresponding statements present the average cluster ratings for participants of the food deserts and food oases. In other words, these columns present the degree of importance of each statement in influencing food buying practices.

Ratings were then grouped into tertiles. Tertile values are based on the degree to which each statement influenced food buying practices and designated as low (1.29–2.50), moderate (2.51–3.72) and high (3.73–4.93). For example, the cluster *Health Consciousness* received an average rating of 3.71 (moderately important for influencing food buying practices) for food desert participants and 4.07 (highly important for influencing food buying practices) for food oasis participants. Overall, the average cluster ratings were similar for residents of the food deserts and food oases. The clusters *Health Consciousness*, *Personal Decisions* and *Shopping Concerns*, were rated higher by participants from the food oases compared to residents of the food deserts. This suggests that food oasis participants perceived the statements within these clusters as more important to influencing food buying practices compared to residents of the food deserts.

**Figure 2:**
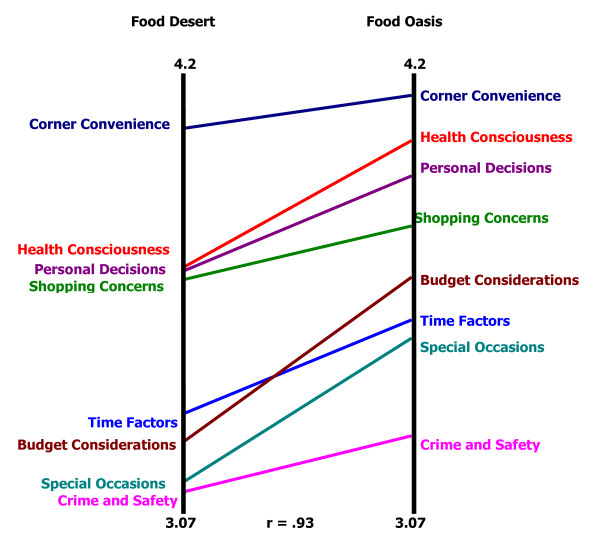
Pattern Match Comparing Factors that Influence Food Buying Practices between Participants from the Food Desert and Food Oasis^*^. ^*^The two vertical lines represent average cluster ratings with results for food desert participants on the left and food oasis participants on the right.

Figure 2 presents a pattern match, a ladder graph representation which illustrates differences in average cluster ratings between food desert and food oasis participants. The vertical black line on the left represents average cluster ratings for food desert participants. The vertical black line on the right represents average cluster ratings for food oasis participants. The 3.07 at the bottom of this line and the 4.2 at the top represent the lowest and highest cluster ratings respectively. These ratings reflect the possible 1 (not at all important) to 5 (extremely important) rating scale participants used to assess how important each statement and subsequent cluster was to influencing food buying practices.

A horizontal rung of the ladder depicts a perfect correlation between the food desert and oasis groups, nearly seen with the cluster *Corner Convenience*. The cluster rating by food desert participants was 4.10 and 4.20 by food oasis participants (ratings not shown). This suggests that both groups perceive the statements within the cluster *Corner Convenience* similarly and as being extremely important to influencing food buying practices. Although the pattern match illustrates similar cluster ratings between the two groups, the average cluster ratings for the food oasis participants were slightly higher. The average cluster ratings and rankings are presented in Table 3. Not only were average cluster ratings similar, but 6 of the 8 clusters have the same rankings for both groups. This finding suggests that both groups perceive the relative order and the degree of importance of each cluster similarly. The Pearson product moment correlation of r = 0.93 at the bottom of the figure represents the correlation between average cluster ratings for all clusters for food desert and food oasis participants. In other words, the correlation of 0.93 suggests that the average cluster ratings are highly correlated between the two groups.

**Table 3:** Average Cluster Ratings for Food Desert and Food Oasis Participants

**Cluster name**	**Food desert rating**	**Food oasis rating**
Health Consciousness	3.71	4.07
Personal Decisions	3.70	3.97
Time Factors	3.29	3.56
Special Occasions	3.10	3.51
Crime and Safety	3.07	3.23
Budget Considerations	3.21	3.68
Shopping Concerns	3.68	3.83
Corner Convenience	4.10	4.20

Statements generated by both food desert and food oasis participants were noted. Examples included “buy in bulk,” “cravings,” “farmer’s markets,” “family influence,” “organic foods,” “quality,” “rodents are common around food,” “store cleanliness,” “taste,” “store hours,” “food pantries,” and “able to buy ethnic foods.” However, few statements unique to each group were documented. Examples of statements unique to food desert participants were “pets need to eat,” “how much I can carry,” “making food stretch,” “food recalls,” and “labels.” Unique statements generated by residents from the food oases included “crime,” “ease of theft,” “use of food with medications,” “24 h stores,” “dollar menus,” and “long lines.”

### Cluster interpretation

**Figure 3:**
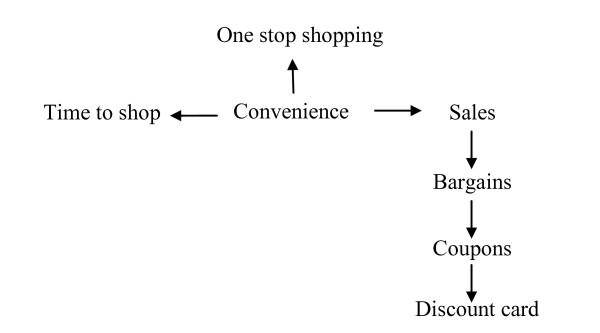
**Interpretation of the Cluster *Corner Convenience *by select food desert participants.**

**Figure 4:**
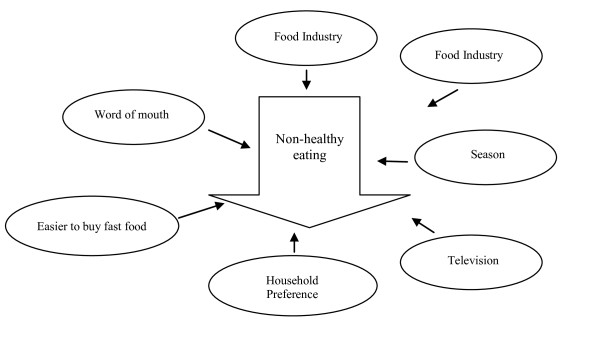
**Interpretation of the cluster *Time Factors *by select food oasis participants. **

The interpretation step of the concept mapping process involved delving deeper into how statements within a cluster were related to each other and how they related back to the focus statement of influencing food buying practices. Participants were divided into smaller groups of 3–4 people and asked to diagram the pathways in which statements within a cluster influence food buying practices. Small groups were asked to illustrate visually how the statements were inter-related. One group diagramed the statements being related using a flow chart. Another group used shapes such as arrows, circles and squares, in a feedback loop, to illustrate how they perceived the clusters being related in influencing food buying practices. The clusters *Corner Convenience* (Figure 3) and *Time Factors* (Figure 4) were selected for interpretation. Clusters that comprise statements that were not easily understood were selected for the in-depth explanation.

#### Corner convenience

*Corner Convenience* was the highest rated cluster for both food desert and food oasis participants. Food desert participants emphasized the statement “one stop shopping” as the central statement within the cluster *Corner Convenience*. As this group stated:

“One stop shopping is important for a group that is missing here, which are men. Guys usually just go to the supermarket and get everything they need, and they don’t generally go to farmer’s markets or specialty stores unless they have to buy a birthday cake or something like that. Even then they go to supermarkets.”

While the statement “one-stop shopping” was described as being the key factor during the interpretation step, this was not reflected in the average statement ratings. “One-stop shopping” was one of two statements within the cluster perceived as moderately important to influencing food buying practices by residents of the food desert. The other 12 statements within the cluster were perceived as being extremely important to influencing food buying practices. In other words, average statement ratings showed statements such as “sales,” “discount card,” “bargains,” and “availability of food” as more important than the notion of “one-stop shopping.”

#### Time factors

The cluster *Time Factors* was the fifth highest rated cluster for food desert participants and the sixth highest rated cluster among food oasis participants. Residents of the food oasis focused on the statement “television” as the key factor in how the statements within the cluster *Time Factors* influence food buying practices. This group explained:

“Advertising always embeds thoughts into your mind. It’s like Doritos and Popeyes [Chicken] sometimes advertise and that gets stuck in your head. I know that’s not healthy eating, but I have a craving for it thanks to my television and the advertising companies behind it.”

Figure 4 illustrates how select food oasis participants perceive the inter-relationship between the statements that comprise the cluster *Time Factors* and how the statements contribute to the consumption of unhealthy foods.

## Discussion

In this study, we explored factors influencing food buying practices among residents of food deserts and oases in Boston. We identified a range of factors that influenced food buying practices, determined the inter-relationship of each factor, and illustrated the pathways. As noted in the Results section, few statements unique to each group (food desert versus food oasis) were identified. Our findings also suggest that few differences in food buying practices among study participants exist regardless of food desert status. One difference was presented by participants residing in the food deserts who explained the statement “how much I can carry.” Participants expressed restrictions in the quantity of food purchased due to the inability to carry heavy bags. Participants explained a reliance on public transportation and difficulty carrying bags on trains and buses and having to walk home from bus stops.

Another difference is in the explanation of the statement “24-hour stores” generated by participants in the food oases. Participants described how the presence of 24-hour stores in their neighborhoods influences food buying practices. Participants who were shift workers described purchasing food from these venues after work as these are the only establishments open in their neighborhood. The purchased food was inevitably energy dense given the availability of nutrient rich foods offered at convenience stores and fast food restaurant drive-throughs [a type of service that allows customers to obtain food without leaving their vehicle] that are open 24-hours each day in the selected neighborhoods.

A novel statement identified that has not been described extensively in the literature was “making food stretch.” Participants of the food deserts described how their desire and intent to make food last longer influenced their food buying practices. For example, some participants received only one paycheck per month, leading to food purchasing on a monthly basis. They bought items that could be broken down into smaller quantities (e.g. packaged meat) for multiple meals, modified by watering down (e.g. sauces), or supplemented by food pantry items.

Another behavior that is not discussed widely in the literature pertains to the statements “crime” and “ease of theft.” These statements, identified by participants in the food oases, were mentioned in the context of fights that erupt in the stores while shopping, crime within neighborhoods, and weighing the risks of stealing when there is not enough money or food vouchers to obtain food. Participants stated that at some supermarkets, patrons could steal up to $250 without being arrested if caught. The disclaimer was that a patron would be arrested if found on the premises after being caught stealing. This was discussed as a viable option for those willing to take the risk.

Existing studies have used either quantitative or qualitative methods to explore facilitators and barriers to healthy eating and food choice. Examples of statements identified that are consistent with those documented in the literature pertained to convenience of getting to the store, store hours, and one-stop shopping [42]; utilizing coupons, or discount vouchers, to defray the cost of a food item [19], having a fixed income has been cited extensively as a factor involved in food buying practices [43]; availability of food [44]; cost of food [17,23]; and transportation options to and from food stores [45,46].

Our findings are in contrast to a study previously conducted by Walker et al. in Pittsburgh, PA [19]. Findings from this study showed that compared to residents of a food oasis, residents of a food desert perceived some factors as more important to influencing food buying practices. The types of factors identified by food desert participants included survival, mental health, and macro-level factors for food desert participants. Food oasis participants uniquely identified conveniences of the food environment and available social service resources [19].

By contrast in Boston, we found few differences in perceptions of factors influencing food buying practices according to food desert status as shown with the pattern match and average cluster ratings (Figure 2). This suggests that food purchasing preferences are similar among residents of low income neighborhoods in Boston irrespective of neighborhood-level access to a supermarket. Additionally, food desert and food oasis participants focused on the importance of emergency food assistance programs. This is consistent to findings from Eikenberry and Smith who found that a promoter of healthy eating among low-income residents was the utilization of federal or local food assistance programs [17]. In our study, participants from both the food deserts and oases used emergency food assistance programs regularly. Participants outlined how they navigated the system of frequenting food pantries and soup kitchens daily. The high dependence and utilization (64.4% of participants) of these resources may account for the observed similarities in average cluster ratings and high correlation coefficient (r = 0.93) However, it can be argued that daily use is no longer considered “emergency” services, but rather chronic use of these resources. Additional studies are needed to implement and evaluate successful city-specific programs that minimize the effects of poor food access to determine how these programs can be adapted to other cities.

A strength of this study was the use of Concept Mapping as the methodology, which is an innovative mixed methods approach to assess residents’ preferences and perceptions. Unlike other qualitative methods such as focus groups or in-depth interviews, concept mapping allows for the inter-relationships between statements to be identified and quantified. These relationships can be used for further theorizing how different factors play a role in influencing food buying practices. A second strength of this study is that we incorporated 4 low-income neighborhoods in a large, urban city.

Limitations of this study were similar to limitations found in qualitative studies, specifically sample size and generalizability. With 67 participants in the study, we are unable to generalize to other food deserts or non low-income food deserts. However, our goal was not to generalize, but to explore perceptions of factors influencing food buying practices so that hypotheses could be generated. Defining a food desert as an area without a supermarket within 0.5 miles of the center of the zip code presents another limitation. Using this definition, a participant could reside in a food desert but live within 0.5 miles of a supermarket in an adjacent zip code, thereby having access to a supermarket. Given the limited resources for this study, we were unable to assess for each potential participant individualized measures of access during participant recruitment. This enhanced method may prove beneficial in a future study. Improved techniques for characterizing areas with differential supermarket access is warranted to provide better agreement in the literature on what constitutes a food desert.

## Conclusions

This study sought to explore food buying practices among low-income residents with differential supermarket access. City-specific measures offered through social service agencies may improve access to foods for residents with poor supermarket access. As a result, food buying practices between residents with and without supermarket access may appear similar. Understanding the availability and utilization of resources within a city that are potentially beneficial to improving food access can help in identifying policies and interventions that can be developed to ultimately promote healthy eating practices.

## Competing interests

The authors declare that they have no competing interests.

## Authors’ contributions

REW, JB and IK contributed to the conception and design of the study. REW facilitated recruitment of participants and was responsible for data collection. JB and IK contributed to questionnaire design. REW and IK drafted the manuscript. All authors read and approved the final version of the final paper.
